# Selective Closed-State Nav1.7 Blocker JZTX-34 Exhibits Analgesic Effects against Pain

**DOI:** 10.3390/toxins10020064

**Published:** 2018-02-02

**Authors:** Xiongzhi Zeng, Pengpeng Li, Bo Chen, Juan Huang, Ren Lai, Jingze Liu, Mingqiang Rong

**Affiliations:** 1 The National & Local Joint Engineering Laboratory of Animal Peptide Drug Development, College of Life Sciences, Hunan Normal University, Changsha 410081, Hunan, China; xiongzhizeng@gmail.com (X.Z.); hnnuchenboo@sina.com (B.C.); HJ875520@163.com (J.H.); 2 The Key Laboratory of Protein Chemistry and Developmental Biology of Ministry of Education, College of Life Sciences, Hunan Normal University, Changsha 410081, Hunan, China; 3 Life Sciences College of Nanjing Agricultural University, 210095, Jiangsu, China; 15062276068@163.com; 4 Key Laboratory of Animal Models and Human Disease Mechanisms of Chinese Academy of Sciences & Yunnan Province, Kunming Institute of Zoology, Kunming 650223, Yunnan, China; 5 Key Laboratory of Animal Physiology, Biochemistry and Molecular Biology of Hebei Province, College of Life Science, Hebei Normal University, Shijiazhuang 050024, Hebei, China

**Keywords:** peptide toxin, sodium channels, Nav1.7, pain, 1. JZTX-34 is a selective peptide toxin on Nav1.7. 2. It binds the domain II voltage sensor of Nav1.7 in a closed configuration. 3. JZTX-34 shows good analgesic effects.

## Abstract

Jingzhaotoxin-34 (JZTX-34) is a selective inhibitor of tetrodotoxin-sensitive (TTX-S) sodium channels. In this study, we found that JZTX-34 selectively acted on Nav1.7 with little effect on other sodium channel subtypes including Nav1.5. If the DIIS3-S4 linker of Nav1.5 is substituted by the correspond linker of Nav1.7, the sensitivity of Nav1.5 to JZTX-34 extremely increases to 1.05 µM. Meanwhile, a mutant D816R in the DIIS3-S4 linker of Nav1.7 decreases binding affinity of Nav1.7 to JZTX-34 about 32-fold. The reverse mutant R800D at the corresponding position in Nav1.5 greatly increased its binding affinity to JZTX-34. This implies that JZTX-34 binds to DIIS3-S4 linker of Nav1.7 and the critical residue of Nav1.7 is D816. Unlike β-scorpion toxin trapping sodium channel in an open state, activity of JZTX-34 requires the sodium channel to be in a resting state. JZTX-34 exhibits an obvious analgesic effect in a rodent pain model. Especially, it shows a longer duration and is more effective than morphine in hot pain models. In a formalin-induced pain model, JZTX-34 at dose of 2 mg/kg is equipotent with morphine (5 mg/kg) in the first phase and several-fold more effective than morphine in second phase. Taken together, our data indicate that JZTX-34 releases pain by selectively binding to the domain II voltage sensor of Nav1.7 in a closed configuration.

## 1. Introduction

Voltage-gated sodium channels (VGSCs) are transmembrane proteins that control the voltage-dependent increase in sodium permeability and play a critical role in initiating and propagating action potentials in excitable cells [1,2]. Nine VGSC α subunit subtypes (Nav1.1–Nav1.9) broadly distributed in the central and peripheral nervous system, skeletal muscle, and the heart have been cloned and identified [3,4]. Many lines of evidence have shown that the subtypes Nav1.3 and Nav1.7–Nav1.9 are involved in pain sensation [5,6,7]. A large number of different mutations increase excitability of Nav1.7 and lead patients to suffer from inherited erythromelalgia (IEM) [8,9]. Recovery from fast inactivation of Nav1.7 is slower than Nav1.3, Nav1.8, and Nav1.9. It also exhibits a slow onset of closed-state inactivation. Those electrophysiological properties of Nav1.7 generate substantial ramp currents in response to slow, small depolarizations [10,11]. That is why Nav1.7 has a profound impact on pain signaling. 

Chronic pain is one of the most difficult clinical problems. As Nav1.7 is considered one of the most attractive targets for relieving chronic pain, scientist have paid a lot of effort to look for a selective inhibitor of Nav1.7. Several small molecules with selectivity on Nav1.7 are currently undergoing clinical development, including PF-05089771 (Pfizer, New York, NY, USA) and CNV1014802 (Convergence Pharmaceuticals, London , UK) [12,13]. Compared to small molecules, peptide toxins have better subtype selectivity due to their larger pharmacophore. Spider venoms are a rich source of ion channel blockers, especially sodium channel toxins. HWTX-IV and ProTx-II are very potent Nav1.7 blockers, which selectively inhibited Nav1.7 by docking on IIS3-S4 linker (site 4) to trap the voltage sensor in a closed state [14,15]. Another Nav1.7 toxin, µ-TRTX-Hhn1b, can efficiently relieve inflammatory pain and neuropathic pain in an animal model [16]. GpTx-1 effectively reduces syndromes of Nav1.7-mediated pain induced by OD1, which is an activator of Nav1.7 [17]. 

In our previous work, JZTX-34 was found to have a selective effect on tetrodotoxin-sensitive (TTX-S) sodium channels from dorsal root ganglia (DRG) neurons. It exhibited no inhibition on tetrodotoxin-resistant (TTX-R) sodium currents [18]. In this study, the analgesic effect and mechanism of JZTX-34 on pain were investigated.

## 2. Results

### 2.1. Synthesis and Refolding of JZTX-34

JZTX-34 was synthesized as a linear peptide using the solid-phase synthesis method. Its molecular weight was 4156.2 Da (Figure S1B), which was the same as the theoretical weight. JZTX-34 was dissolved in a refold buffer (100 mM Tris-HCl, 100 mM NaCl, 1 mM GSSG, 10 mM GSH) to form the same structure as native JZTX-34. As shown in Figure S1A, linear JZTX-34 was eluted at 52.1 min (red arrow), and the fold peptide was eluted at 53.9 min (blue arrow). JZTX-34 and the matrix were mixed together at a ratio 1:1 and then spotted onto a matrix-assisted laser desorption ionization time-of-flight (MALDI-TOF) plate. Spots were analyzed by an UltraFlex I mass spectrometer in a positive ion mode to determine the molecular weight. The molecular mass of refold JZTX-34 was 4150.0 Da (Figure S1C), which was 6 Da less than that of the linear peptide, indicating that three disulfide bonds were formed.

### 2.2. Inhibition of JZTX-34 on Rat DRG Sodium Channels

**Figure 1:**
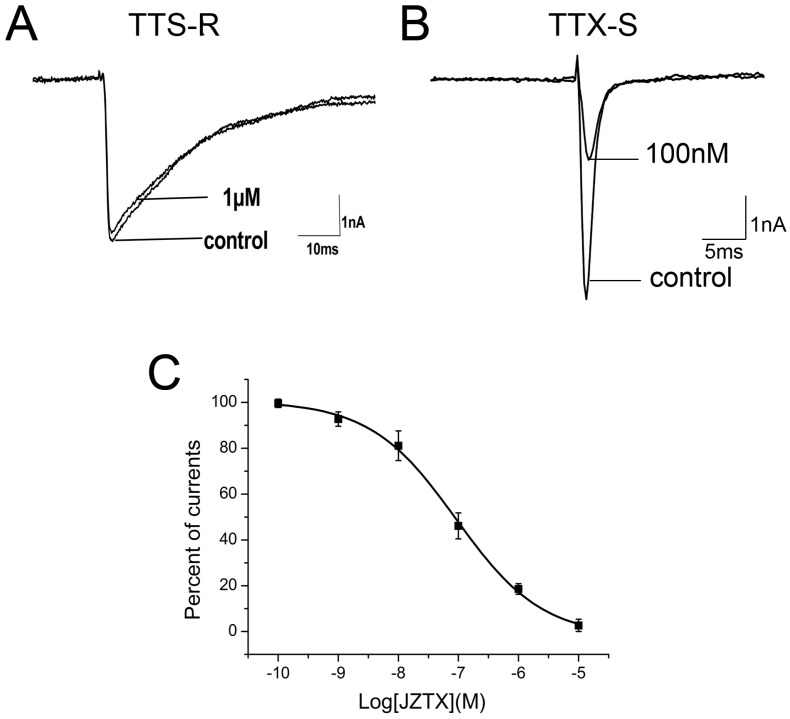
Effects of synthetic JZTX-34 on TTX-S and TTX-R sodium channels from rat DRG neurons. Inward currents traces were induced by a 50 ms depolarizing potential to −10 mV from a holding potential of −80 mV every 5 s. (**A**) Effect of 1μM synthetic JZTX-34 on TTX-R currents. (**B**) Effect of 1 μM synthetic JZTX-34 on TTX-S currents. (**C**) Dose-dependent inhibition of synthetic JZTX-34 on TTX-S sodium channels (*n* = 5). Data points (mean ± S.E.) were fitted according to the standard Hill equation.

In our previous work, we have observed that 1 µM native JZTX-34 could inhibit TTX-S currents on DRG neurons. In this study, effects of synthetic JZTX-34 on TTX-S sodium channel were investigated to confirm its activity. Using the patch-clamp technique in whole cell configuration, sodium channel currents were elicited by a 50-ms depolarizing potential to −10 mV from a holding potential of −80 mV. TTX-resistant (TTX-R) sodium channels were isolated by adding 100 nM TTX into an external solution to inhibit TTX-sensitive currents on small-sized DRG neurons (<20 µm). One µM synthetic JZTX-34 inhibited ~81% TTX-S sodium currents (Figure 1B) and had no effect on TTX-R sodium currents (Figure 1A). The IC_50_ value was yielded to be 91 nM (Figure 1C), which was close to that of the native toxin (84 nM). These data suggested that native and synthetic JZTX-34 might have the same biological activities.

### 2.3. Subtype Selectivity of JZTX-34 Interaction with Sodium Channels

**Figure 2:**
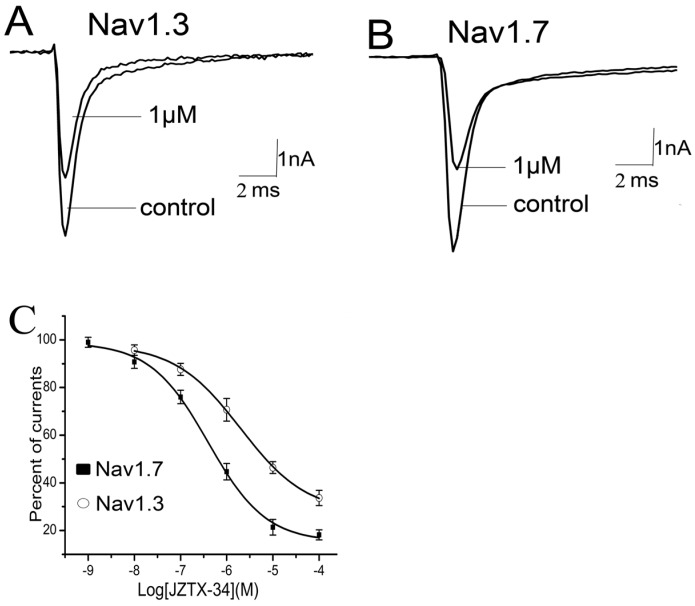
Selectivity of JZTX-34 on sodium channel subtypes. All inward sodium channels were elicited by a 50 ms depolarizing potential to −10 mV from a holding potential of −80 mV every 5 s. One μM JZTX-34 was added to rNav1.3. (**A**) hNav1.7. (**B**,**C**) Dose-dependent inhibition of JZTX-34 on rNav1, rNav3, and hNav1.7 (*n* = 5). Data points (mean ± S.E.) were fitted according to the standard Hill equation.

Nine sodium channel subtypes have been identified and classified as TTX-S (Nav1.1–Nav1.4, Nav1.6, and Nav1.7) or TTX-R types (Nav1.5, Nav1.8, and Nav1.9). JZTX-34 can selectively inhibited the TTX-S sodium channel. However, the subtype selectivity of JZTX-34 on the subtype of sodium channel is still unknown. In this work, effects of JZTX-34 on VGSC subtypes (Nav1.1–Nav1.8) were examined. Nav1.1–Nav1.7 were transfected into HEK293 cells and Nav1.8 currents were expressed in ND cells. Inward sodium currents were elicited by a 50 ms depolarizing potential to −10 mV from a holding potential of −80 mV every 5 s. As seen in Figure 2, 1 µM JZTX-34 can reduce currents of Nav1.3 and Nav1.7 by 29.4 ± 4.6% and 54.6 ± 5.3%, respectively (Figure 2A,B). No effects on other sodium channel subtypes were found (Nav1.1, Nav1.2, Nav1.4–Nav1.6, and Nav1.8) (Figure S2). The inhibition of Nav1.3 and Nav1.7 was concentration-dependent, and the IC_50_ values were evaluated to be 7950 nM and 610 nM, respectively (Figure 2C). Our data suggest that JZTX-34 preferentially inhibited Nav1.7 than other sodium channel subtypes. 

### 2.4. Kinetics Effects of JZTX-34 on Nav1.7

It has been demonstrated that JZTX-34 selectively acted on Nav1.7. We investigated the effects of this toxin on the gating properties of Nav1.7. The current-voltage relationship of Nav1.7 was investigated using step depolarization ranging from −80 to +80 mV from a holding potential of −80 mV. Under control conditions, the threshold of initial activation was about −40 mV, and the inward current peaked at −20 mV. The treatment of 1 µM toxin did not alter the current-voltage relationship of Nav1.7 (Figure S3A). The midpoint of channel activation before and after application of 1 µM toxin was −27.9 ± 3.3 and −28.1 ± 2.9 mV, respectively (Figure S3B). It was suggested that JZTX-34 did not alter the voltage dependence of activation. The effect of JZTX-34 on steady-state inactivation was also investigated using a standard two-pulse protocol as described in the legend. As seen in Figure S3C, the estimated midpoints of steady-inactivation with or without the presence of 1 µM toxin of Nav1.7 were about −67.3 mV and −73.7 mV, respectively, indicating that JZTX-34 caused a small hyperpolarizing shift in the steady-state inactivation of Nav1.7 (Figure S3C).

### 2.5. Binding Site of Toxin on Nav1.7

**Figure 3:**
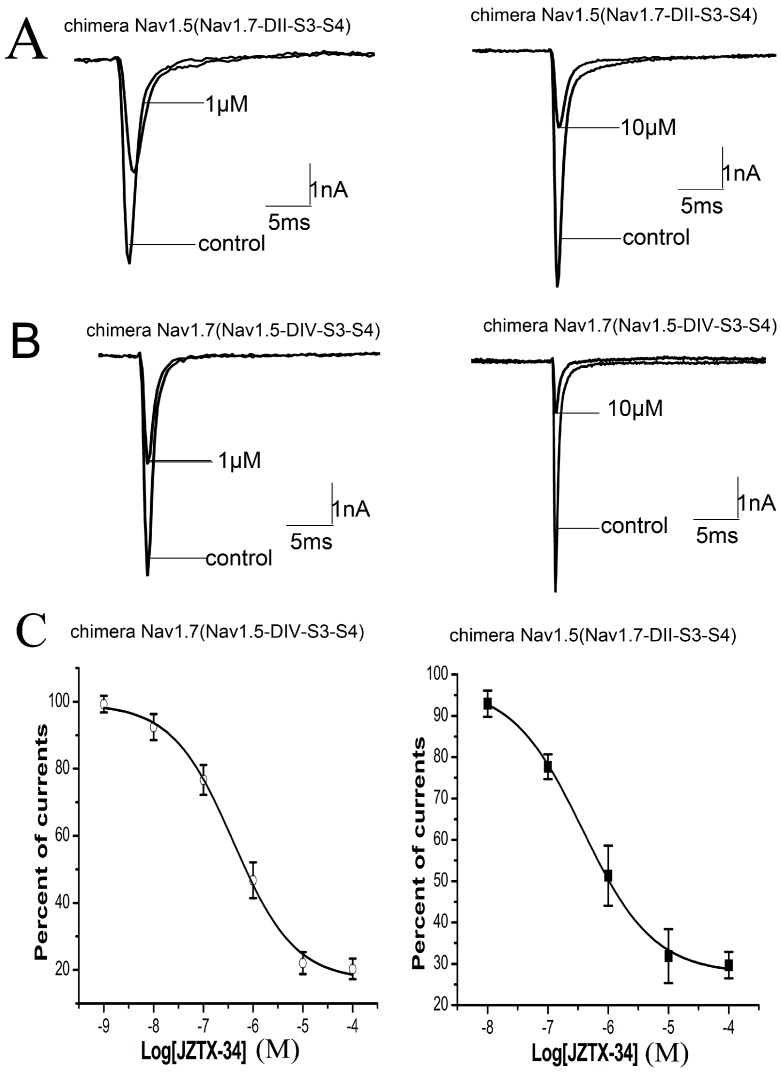
JZTX-34 inhibited sodium channel subtypes Nav1.7 by binding to DII S3-S4. (**A**) Inhibition of 1 μM and 10 μM toxins on chimeric Nav1.5 (Nav1.7-DII-S3-S4). (**B**) Inhibition of 1 and 10 μM toxins on chimeric currents of Nav1.7 (Nav1.5-DIV-S3-S4) sodium currents. (**C**) Dose-dependent inhibition of synthetic JZTX-34 on Nav1.7 (Nav1.5-DIV-S3-S4) and (Nav1.7-DII-S3-S4) (*n* = 5). Data (mean ± S.E.) were fitted according to the standard Hill equation.

Many animal toxins alter activation or inactivation of sodium channels by binding to the S3–S4 regions of channels, which are probably the external linkers or voltage sensors of sodium channels. α-Scorpion toxins slow down fast-inactivation of sodium channels by binding to the extracellular linker loop of DIVS3-S4 (neurotoxin site 3) [19]. Meanwhile, β-scorpion toxins that interact with DIIS3-S4 linker enhance the activation of sodium channels by trapping DII voltage sensor in an activated state [19,20]. As Nav1.5 channel is resistant to the inhibition of JZTX-34, we investigated the binding site of JZTX-34 to Nav1.7 by constructing chimeric channels between Nav1.5 and Nav1.7. In chimera #1, the DIIS3-S4 linker of Nav1.5 was replaced by the corresponding part of Nav1.7. In chimera #2, the DIVS3-S4 linker of Nav1.7 was substituted by the corresponding part of Nav1.5. Inward sodium currents of the two chimeric channels were elicited by a 50 ms depolarizing potential of −10 mV from a holding potential to −80 mV every 5 s. As shown in Figure 3, 1 µM JZTX-34 inhibited the currents of chimera #1 by 47.6 ± 3.6% (Figure 3A). When the concentration increased to 10 µM, the current amplitude of chimera #1 were depressed by 79.5 ± 2.8% (Figure 3A,). The IC_50_ value was 706.3 nM to chimera #1 (Figure 3C), which was almost the same to the value on WT Nav1.7 (610 nM). JZTX-34 at 1 μM and 10 µM could inhibit chimera #2 currents by 43.6 ± 4.5% and 76.3 ± 3.6%, respectively (Figure 3B, *n* = 5). The IC_50_ value on chimera #2 was 1.05 µM (Figure 3B), closing to WT Nav1.7. It has been demonstrated that JZTX-34 did not show any activity on Nav1.5, but the sensitivity of this toxin to Nav1.5 was extremely enhanced after DIIS3-S4 linker of Nav1.7 transplanted into Nav1.5 (chimera #1). This suggested that JZTX-34 may interact with Nav1.7 by binding to DIIS3-S4 linker. Chimera #2 did not change JZTX-34’s sensitivity of Nav1.7, implying DIVS3-S4 linker of Nav1.7 is not the bind site of JZTX-34.

**Figure 4:**
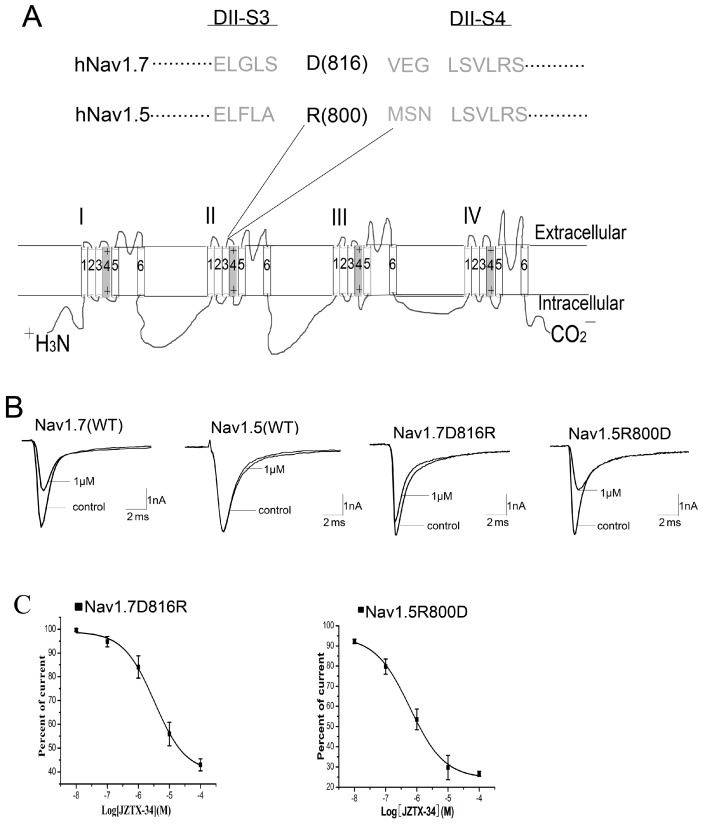
Effects of JZTX-34 on mutants of Nav1.7 and Nav1.5. (**A**) Amino acid sequence alignment of DII S3-S4 linker of Nav1.5 and Nav1.7 and schematic diagram of sodium channel α subunit. The positions of amino acids of interest are shaded in bold black. (**B**) Inhibition of WT Nav1.7, WT Nav1.5, Nav1.7 (816R) and Nav1.5 (R800D) by 1 µM JZTX-34. (**C**) Dose-dependent inhibition of JZTX-34 on Nav1.7 (D816R) and Nav1.5 (R800D) (*n* = 5). Data points (mean ± S.E.) were fitted according to the standard Hill equation.

We next investigated the critical residues in DIIS3-S4 linker that determined the sensitivity of Nav1.7 to JZTX-34. Sequence alignment of DIIS3-S4 linker of Nav1.5 and Nav1.7 is shown in Figure 4A. An acidic residue, D816, is present in the DIIS3-S4 linker of Nav1.7, while the corresponding residue in Nav1.5 is an alkaline amino acid, R800. To determine whether the acidic residue D816 occupies an important role in toxin binding, we constructed two mutants: D816R of Nav1.7 and R800D of Nav1.5. One µM of JZTX-34 inhibited 54.3 ± 5.2% of WT Nav1.7 currents (Figure 4A), but only 16.8 ± 4.6% currents of D816R were inhibited at the same concentration (Figure 4B). The IC_50_ value of Nav1.7 D816R was 19.5 µM (Figure 4C), suggesting that D816R decreased toxin binding affinity to Nav1.7 by ~32 folds. To further confirm the key role of D816R in blocking Nav1.7 by JZTX-34, we examined the effect of JZTX-34 on Nav1.5 mutant R800D. In contrast to WT Nav1.5 resistant to JZTX-34 (Figure 4C), 1 μM JZTX-34 could obviously inhibited the currents of Nav1.5 R800D, and the IC_50_ value was 1.26 µM (Figure 4C). Thus, the acidic residue D816 in DIIS3-S4 linker may be critical for the sensitivity of Nav1.7 to JZTX-34.

### 2.6. JZTX-34 Binds to Voltage Sensor of Domain Ii in A Closed Configuration

**Figure 5:**
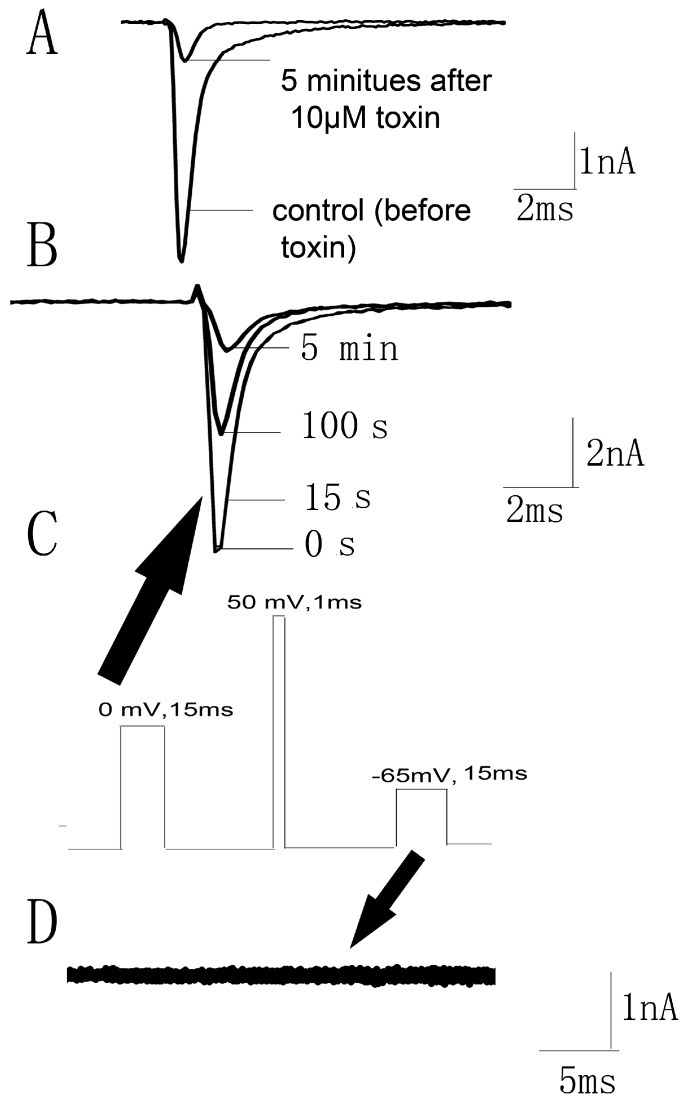
JZTX-34 traps the domain II voltage sensor in closed configuration. (**A**) Nav1.7 current traces were elicited by a protocol as following. First, an inward sodium currents were elicited by a 50 ms depolarizing potential to 0 mV from a holding potential of −80 mV every 5 s. Second, 10 μM JZTX-34 were added to bath solution. Finally, the same pulse protocol was applied to elicit inward currents after a 5 min toxin treatment. (**B**) The current traces 0, 15 ms, 100 ms, and 5 min after toxin treatment are shown, which were elicited by a protocol as shown in C. (**C**) This pulse protocol was included three phases and applied every 5 s. In the first pulse, a 15 ms moderate depolarizing potential to 0 mV from a holding potential of −80 mV was used to induce sodium currents. After 61.2 ms of first pulse, a 1 ms strong conditioning depolarizing potential to 50 mV from a holding potential of −80 mV was applied. In the third phase, a second period of 61.2 ms at the holding potential of −80 mV, a 15ms test pulse to −65 mV was given again. (**D**) The current traces after toxin treatment were elicited by the third pulse (black arrow) of protocol C.

It has been proven that scorpion β-toxins trapped the domain II S4 voltage sensor in the outward position [10]. However, HWTX-IV inhibited sodium channels by trapping voltage sensor in a closed configuration [15]. We wondered whether JZTX-34 blocked sodium channels in a resting state or an outward position. The current traces were recorded by a 50 ms depolarizing pulse of −10 mV from a holding potential of −80 mV. At first, 10 µM JZTX-34 was added to cells and incubated for 5 min. Then, the currents were elicited by the protocol described before [21]. It was obviously seen that currents were significantly inhibited at the first pulse (Figure 5A), implying that JZTX-34 might bind the Nav1.7 channel in a resting state. To further confirm this, the same protocol described by Cestele et al. [21] was used to assess whether JZTX-34 traps the domain II S4 voltage sensor of the WT Nav1.7 in a closed position. A 15 ms test pulse to 0 mV was applied from a holding potential of −80 mV and was followed by a 1 ms conditioning pulse to +50 mV. Then, a 15 ms test pulse to −65 mV was used to detect whether the currents were elicited. In the presence of 10 µM JZTX-34, the current amplitude induced by the first test pulse was greatly depressed in a time-dependent manner (Figure 5B), and no inward current was observed after the second test pulse of −65 mV (Figure 5D). In contrast to JZTX-34, Css IV could induce an inward current at −65 mV because it could trap the voltage sensor in an outward position [21]. This indicates that JZTX-34 does not trap the domain II S4 in an outward position but may trap the domain II voltage sensor in a resting state.

### 2.7. Analgesic Effects of JZTX-34 on Pain

**Figure 6:**
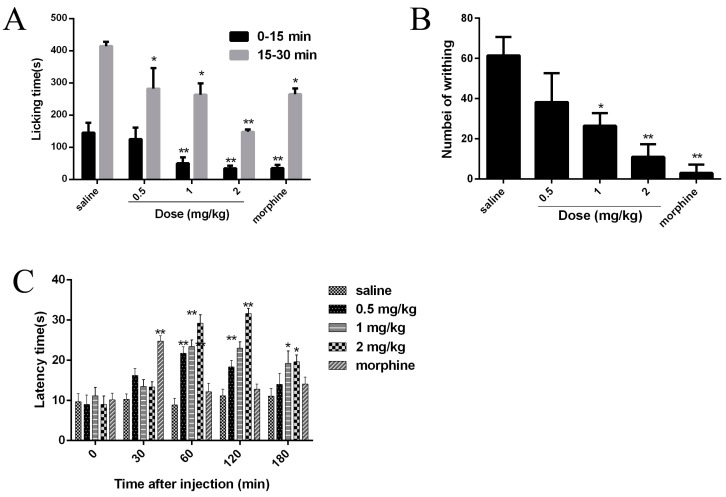
Effects of JZTX-34 on pain. (**A**) Effects of JZTX-34 on the early phase (0–5 min) and late phase (15–30 min) of formalin-induced paw licking response in mice. (**B**) Effects of JZTX-34 on the acetic acid-induced writhing response in mice. (**C**)Effects of JZTX-34 on the hot plate test in mice. All data points are shown as mean ± S.E. Six animals were used for each separate group experiment. * *p* < 0.05, ** *p* < 0.01 significantly different results compared to the saline group.

Since Nav1.7 plays an important role in pain, the analgesic effects of JZTX-34 were examined in three animal models (formalin-induced paw licking, acetic acid-induced writhing, and hot plate). Intraplantar injection of formalin causes pain and can be divided into two phases. The early phase was neurogenic pain and usually occurred within 0–15 min), and the late phase was inflammatory pain lasting from 15 to 30 min). In the control mice group, paw licking time was 145.5 s in the early phase and 414.5 s in the late phase (Figure 6A). In the early phase, the paw licking times were 125.5 s, 50.1 s, and 34.7 s for 0.5 mg/kg, 1 mg/kg, and 2 mg/kg JZTX-34, respectively, while the numbers were 35.3 s after injection of 5 mg/kg morphine (Figure 6A). In the late phase, the paw licking times were reduced to 282.2 s, 263.6 s, and 148.0 s for mice treated with 0.5 mg/kg, 1 mg/kg and 2 mg/kg JZTX-34, respectively, while the values were decreased to 265 s after injection of 5 mg/kg morphine (Figure 6A).

Abdominal contraction and hind limb stretching were induced by intraperitoneal injection of 0.8% (*v*/*v*, 10 mL/kg) acetic acid. JZTX-34 (0.5, 1 or 2 mg/kg) or morphine (5 mg/kg) were immediately intraperitoneally (i.p.) injected into mice after intraperitoneal injection of acetic acid. The negative control group received the same volume of the vehicle. In the acetic acid-induced writhing test, intraperitoneal injection of 0.5 mg/kg, 1 mg/kg, and 2 mg/kg JZTX-34, the number of writhing reduced from 61 to 38, 26, and 11, respectively. The injection of 5 mg/kg morphine caused a reduction to 3 (Figure 6B).

Mice with a latency time about 10 s were selected for the hot plate test. JZTX-34 (0.5, 1, or 2 mg/kg) or morphine (5 mg/kg) were dissolved in vehicle (0.9% saline) and then intraperitoneally (i.p.) injected into the mice. After administration of test samples, the latency time was recorded at the time points of 30 min, 60 min, 120 min, and 180 min (Figure 6C). Negative control animals received the same volume of vehicle. At 30 min, morphine (5 mg/kg) showed the strongest analgesia effect on the hot plate model with a latency increase to 24.8 (Figure 6C). Then the analgesia significantly decreased to 12.2 s at the time of 60 min. JZTX-34 showed a longer and stronger analgesia effects than morphine. The most attractive analgesia effects were observed at 120 min. At that time, JZTX-34 at dose of 0.5, 1, or 2 mg/kg increased the latency time to 18.3 s, 23.0 s, and 31.6 s, respectively (Figure 6C).

## 3. Discussion

Venomous animals have developed a complex combinational peptide library of ion channel toxins and other peptides or proteins in their venom to diversify their toxin weapons [22]. Peptide toxins acting on voltage-gated sodium channels (VGSCs) derived from spider venoms are considered as essential molecular probes for studying the distribution, physiological roles, and structure-function relationship of VGSCs and lead molecules for the development of novel drugs [23]. 

Many peptide toxins, which alter gating properties of VGSCs, have been identified to trap the DIIS3-S4 linker of sodium channels [24]. However, the molecular mechanisms of inhibition of sodium currents by peptide toxins are greatly divergent. The classical site 4 toxins, e.g., scorpion β-toxins Css IV, impair the activation of Nav1.2 sodium channels and the binding the voltage sensor in the open configuration [21,25]. Another site 4 toxin, HWTX-IV f, blocks Nav1.7 and traps the DII S3-S4 linker in a closed configuration without affecting activation or inactivation of channels [25]. ProTx-II, isolated from the venom of the trarantula *Thrixopelma pruriens*, also binds to domain II of the Nav1.7 voltage sensor in a closed configuration. Unlike HWTX-IV, ProTx-II modified the voltage dependence of VGSC inactivation [26]. In this study, we found that JZTX-34 inhibited sodium channel subtype Nav1.7 by binding to neurotoxin receptor site 4 in the closed configuration. Meanwhile, JZTX-34 caused a small hyperpolarizing shift in the steady-state inactivation of the Nav1.7 channel. The mechanism of JZTX-34 acting on Nav1.7 exhibited little difference to HWTX-IV and ProTx-II. Although HWTX-IV and ProTx-II both bind to the S3–S4 linker of domain II, the specific binding residues are different. E818 is the crucial residue for HWTX-IV to interact with Nav1.7, while the crucial residue of ProTx-II binding to Nav1.7 is F813 [27]. JZTX-34 selectively acts on Nav1.7 and shows no effect on Nav1.5. When the DIIS3-S4 linker of Nav1.7 moved to Nav1.5, the sensitivity of Nav1.5 to JZTX-34 extremely increased. A mutation D816 in DIIS3-S4 linker of Nav1.7 significantly decreased binding affinity of Nav1.7 to JZTX-34. Interestingly, the reverse mutant R800D a in Nav1.5 greatly increased its binding affinity to JZTX-34. Thus, D816 is the crucial residue for JZTX-34 to interact with Nav1.7. 

Since Nav1.7 in the peripheral nervous system is a promising therapeutic target for pain, the search for novel Nav1.7 inhibitors attract significant attention. Venom toxins acting on Nav1.7 have been identified as promising therapeutic leads because they are generally more potent and selective than small molecules. When ProTx-II was applied to desheathed cutaneous nerves, it completely blocked the C-fiber compound action potential and exerted a strong analgesic effect on severe pain [27]. HWTX-IV efficiently eased the acute inflammatory pain and chronic neuropathic pain [28]. The two toxins also show activity on other sodium channel subtypes. Low selectivity limits it use as analgesic. Other toxins such as GpTx-1 and CcoTx1 were engineered in order to improve potency, selectivity, stability, and bioavailability of venom-derived lead peptide compounds targeting Nav1.7 [29,30]. JZTX-34 shows moderate activity on Nav1.7 without any effects on Nav1.1, Nav1.2–Nav1.6, and Nav1.8. Besides, the IC_50_ value of JZTX-34 to Nav1.7 is about 20-fold lower than that to Nav1.3, indicating that JZTX-34 preferentially acts on Nav1.7. The selectivity on Nav1.7 implies that JZTX-34 could be a good candidate drug to treat pain. Consistent with this proposal, JZTX-34 has proven to be an effective analgesic in rodent pain models (formalin-induced, acid-induced, and thermal models). In the formalin-induced pain model, JZTX-34 at a dose of 2 mg/kg is equipotent with morphine (5 mg/kg) in the first phase and several-fold more effective than morphine in second phase. In thermal model, JZTX-34 (2 mg/kg) shows a longer and stronger analgesia effects than 5 mg/kg morphine. 

Although there are many spider toxins with activity on the sodium channel, few spider toxins show selectivity on Nav1.7. In this study, we found that JZTX-34 exhibits selectivity activity on Nav1.7 and binds to D816 in DIIS3-S4 linker of Nav1.7 in a closed configuration. Moreover, it shows a longer duration to and is more effective than morphine in rodent pain models. Thus, JZTX-34 is a promising lead molecule for future clinical development for novel therapeutics in the treatment of pain.

## 4. Experimental Section

### 4.1. Toxin and Animals

The synthesized JZTX-34 was obtained according to the procedure as previously described [31]. The purity of reduced peptides was determined to be more than 95% using high-performance liquid chromatography and MALDI-TOF mass spectrometry. Rats (Sprague-Dawley) and Kunming mice were maintained in the Laboratory of Protein Chemistry of Hunan Normal University. All of the experimental involving animals were approved by the Animal Care and Use Committee of Hunan Normal University (2016-273, 13 October 2016).

### 4.2. Peptide Synthesis and Refolding

A Boc protection strategy was used to synthesize the linear JZTX-34. After synthesis, 0.1 mg peptide were dissolved into 1 mL refold buffer containing 50 mM Tris-Hcl, 50 mM NaCl, 0.10 mM GSSG, 1 mM GSH, and pH7.2. JZTX-34 in buffer was kept at a temperature of 25 °C for 24 h. TFA was added to the refold buffer in order to terminate the reaction. After refolding reaction, the oxidation toxin was loaded onto an analytical C18 reversed-phase (RP) HPLC column in an analytical RP-HPLC. The molecular weight and purity of the JZTX-34 was determined using MALDI-TOF.

### 4.3. Mass Spectrometric Analysis

Linear and refold JZTX-34 were dissolved in 0.1% (*v*/*v*) trifluoroacetic acid/water. The matrix used for matrix-assisted laser desorption ionization time-of-flight (MALDI-TOF) was α-cyano-4-hydroxy-cinnamic acid (CCA, saturated solution in 60% ACN: 0.1% TFA). Then, a 0.5 µL sample was spotted onto a MALDI-TOF plate with 0.5 µL matrix. Molecular weight of JZTX-34 was analyzed by an UltraFlex I mass spectrometer (Bruker Daltonics, Bremen, Germany) in a positive ion mode. Mass spectra were recorded according to the manufacturer’s instruction.

### 4.4. Acute Isolation of DRG Neurons and Sodium Channel Recording

Sprague-Dawley rats about 200 g of either sex were decapitated. Then the DRG were quickly separated from spinal cord of each rat [32]. Cells dissociated from DRG were suspended in Dulbecco’s modified Eagle’s medium. DNase (0.1 g/L, type III), collagenase (1.0 g/L, type IA), and trypsin (0.5 g/L, type III) were added to the medium and then incubated at 34 °C for 30 min to obtain single cells. Trypsin inhibitor (1.5 g/L, type II-S) was used to terminate the reaction after incubation for 30 min. Acutely dissociated DRG neurons were transferred into 35-mm culture and maintained at 34 °C with 5% CO_2_ for 3 h. Whole-cell patch clamp recordings were performed using Axon Multiclamp 700B amplifier (Molecular Devices, Sunnyvale, CA, USA). The P/4 protocol was used to subtract linear capacitive and leakage currents. The standard pipet solution for sodium current recordings on DRG cells contained (in mM): 105 CsF, 35 NaCl, 10 HEPES, and 10 EGTA at pH 7.4. The bath solution for sodium channel on DRG cells contained (in mM): 150 NaCl, 2 KCl, 5 D-glucose, 1 MgCl_2_, 1.5 CaCl_2_, and 10 HEPES at pH 7.4. 

### 4.5. Construction of Chimeric and Mutants of Nav1.5 and Nav1.7

cDNA genes encoding rat Nav1.1, Nav1.2, Nav1.3, Nav1.4, Nav1.5, human Nav1.7 (hNav1.7), and hNav1.8 were subcloned into the vectors pRGB4 and pcDNA3.1-mod, respectively. The chimera of Nav1.5 (Nav1.7-DII-S3-S4) and Nav1.7 (Nav1.5-DIV-S3-S4) were constructed according the methods described previously [21]. Mutations of Nav1.5 and Nav1.7 were constructed using QuikChange II XL Site-Directed Mutagenesis kit (Agilent Technologies Inc, Palo Alto, CA, USA). All chimera and mutations were sequenced to determine the right sequences.

### 4.6. Transient Expression of Sodium Channel Subtypes

The HEK293T cells were maintained in standard culture conditions (5% CO_2_ and 37 °C). Then, 10% fetal bovine serum was added to Dulbecco’s modified Eagle’s medium. Sodium channel subtypes (Nav1.1–Nav1.5, Nav1.7), the chimera and mutants of Nav1.5 and Nav1.7 with β1subnit tagged with eGFP in a molar ratio of 2:1, were transiently co-transfected into HEK293 cells using Lipofectamine 3000 (Invitrogen, Carlsbad, CA, USA). Nav1.8, β1subnit, and eGFP were co-transfected into ND cells using the same protocol. After culturing for 36–72 h, cells with obvious green fluorescence were selected for sodium currents recordings the internal solution for sodium currents recording that contained (in mM) 140 CsF, 5 NaCl, 10 HEPES, 3 Na_2_-ATP, and 10 EGTA. The external solution contained (in mM) 150 NaCl, MgCl_2_ 1, 5 KCl, 10 glucose, 1 CaCl_2_, and 10 HEPES.

### 4.7. Whole-Cell Patch-Clamp Recordings 

Sodium currents were recorded from DRG, HEK293, and ND cells by the whole-cell patch-clamp technique at 25 °C. The patch pipettes resistances were controlled at 2–3 mΩ by adjusting the pulling temperature with a two-stage vertical microelectrode puller (PC-10, Narishige, Tokyo, Japan). Then, the patch pipettes were fire-polished by a heater (Narishige, Tokyo, Japan) for further use. Experiments date were acquired from whole-cell Axon 700B patch clamp amplifier (Molecular Devices, Sunnyvale, CA, USA) and analyzed by the program Clampfit10.0 (Molecular Devices, Sunnyvale, CA, USA) and Sigmaplot. Linear capacitive and leakage currents were subtracted by P/4 protocol.

### 4.8. Paw Licking Induced by Formalin

Formalin test was performed according to the method described previously [33]. JZTX-34 and morphine were dissolved in 100 μL saline and intraperitoneally (i.p.) injected into mice. After test samples were intraperitoneally (i.p.) injected, 5% formalin (20 μL) was injected to the plantar surface of right hind paw. The time spent licking the injected paw was counted every 5 min. Phase I was defined as 0–10 min, and Phase II defined as 15–35 min. 

### 4.9. Abdominal Writhing Response Caused by Acetic Acid 

Acetic acid (200 μL 0.8% *v*/*v*) were intraperitoneal injected into mice. Then, 100 μL saline containing JZTZ-34 and morphine were immediately intraperitoneally (i.p.) injected [34]. The same volume of saline were also intraperitoneally (i.p.) injected into mice in the control group. The abdominal writhing responses after acetic acid injection were continuously counted for 30 min.

### 4.10 Hot Plate

Mice were put on a hot plate apparatus (model HZ66-ZH-YLS-6B, Shanghai, China) in order to test response time to a thermal stimulus. The responses time of mice at about 10 s was selected for the test. After the injection of JZTX-34 and morphine, measurements were immediately started. Hind paw licking or jumping were regarded as a sign to avoid heat nociception. 

### 4.11 Data Analysis

Data from Axon 700B patch clamp were analyzed by Clampfit10.0 (Molecular Devices, Sunnyvale, CA, USA). Dose-response curves were fitted by the Hill logistic equation: *y* = 1 − (1 − *fmax*)/(1/([*Tx*]/*IC*_50_)*^n^*). In this equation, n stands an empirical Hill coefficient and fmax is the fraction of current resistant to inhibition at high toxin (*Tx*) concentration. The *G-V* curves were obtained by calculating the conductance (*G*) at each voltage (*V*) using the equation *G = I/(V*1 − *V_rev_)*, with *V_rev_* being the reversal potential determined for each cell individually. *G-V* curves were fitted using a Boltzmann equation—*y* = 1/(1 + *exp*[*(V_a_* − *V)*/*κ*])—in which *V_a_*, *V*, and *κ* represent midpoint voltage of kinetics, test potential, and slope factor, respectively. All the results from patch clamp and animal models are expressed as means ± standard error of the mean (SEM). Statistical significance was calculated using Student’s t-test and was defined as *p* < 0.05. 

## Supplementary Materials

The following are available online at http://www.mdpi.com/2072-6651/10/2/64/s1, Figure S1: Identification of linear and refolded JZTX-34, Figure S2: 1 μM JZTX-34 showed no effect on Nav1.1, 1.2, 1.4, 1.5, 1.6 and 1.8, Figure S3: Effects of JZTX-34 on the current-voltage relationship and the steady-activation and inactivation of sodium channel subtypes Nav1.7.
