# Supplementary material for: Selective Closed-State Nav1.7 Blocker JZTX-34 Exhibits Analgesic Effects against Pain
Source: Toxins (Basel). 2018 Feb 2;10(2):64. doi: 10.3390/toxins10020064 (PMC5848165; doi:10.3390/toxins10020064)
Supplement: Supplementary file 1 [file toxins-10-00064-s001.pdf]

Supplementary Materials: Selective Closed-State Nav1.7 Blocker JZTX-34 Exhibits Analgesic Effects against Pain

Xiongzhi Zeng, Pengpeng Li, Bo Chen, Juan Huang, Ren Lai, Jingze Liu, and Mingqiang Rong

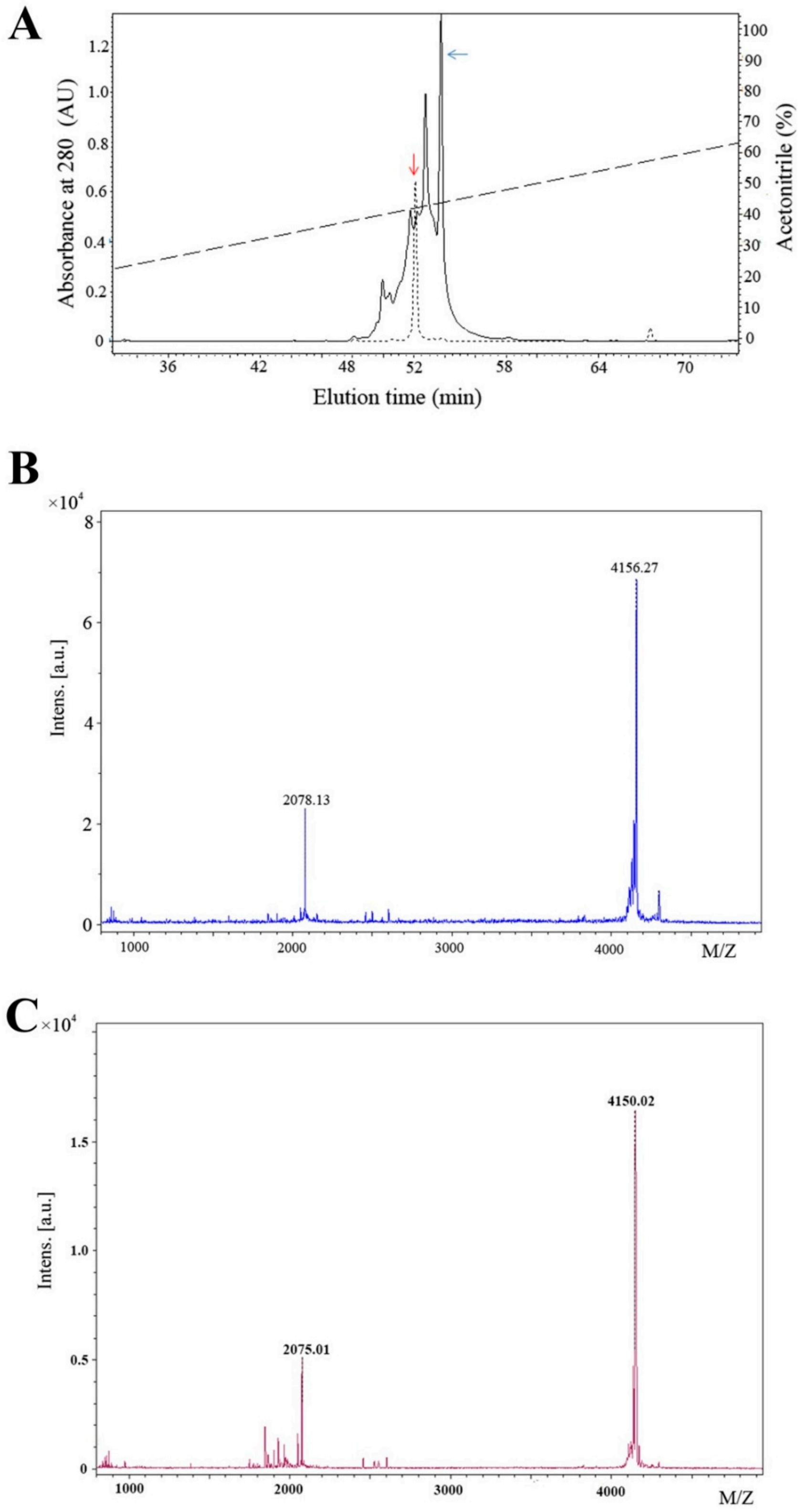

Figure S1. Identification of linear and refolded JZTX-34. (**A**) Linear and refolded JZTX-34 was separated by analytical reverse-phase HPLC (monitored at 280 nm). (**B**) MALDI-TOF mass spectra of synthetic JZTX-34. (**C**) MALDI-TOF mass spectra of refold JZTX-34.

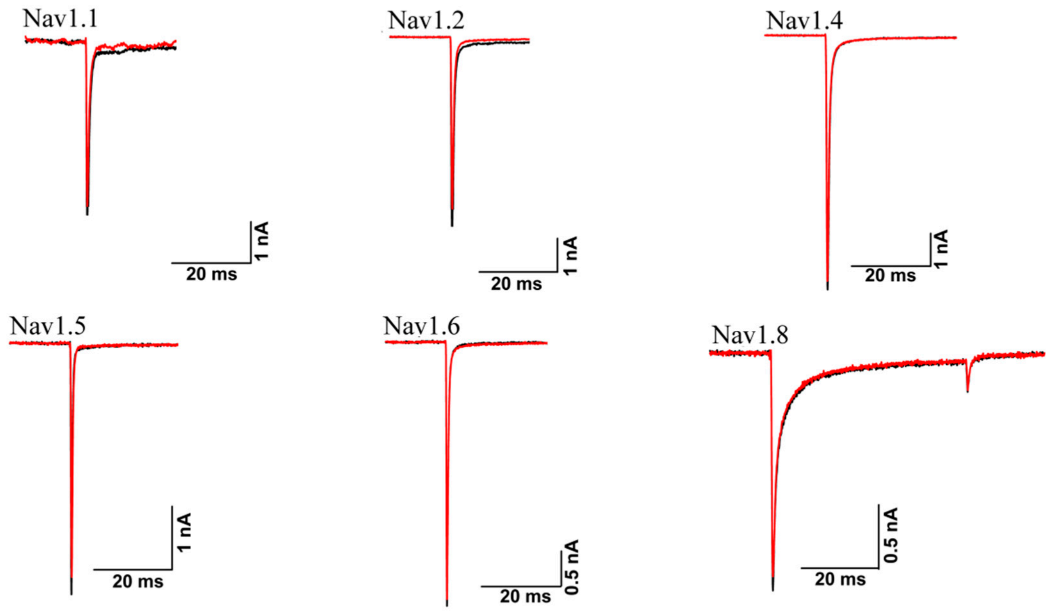

Figure S2. 1 μM JZTX-34 showed no effect on Nav1.1, 1.2, 1.4, 1.5, 1.6 and 1.8. All inward sodium channels were elicited by a 50 ms depolarizing potential of −10 mV from a holding potential of −80 mV every 5 s.

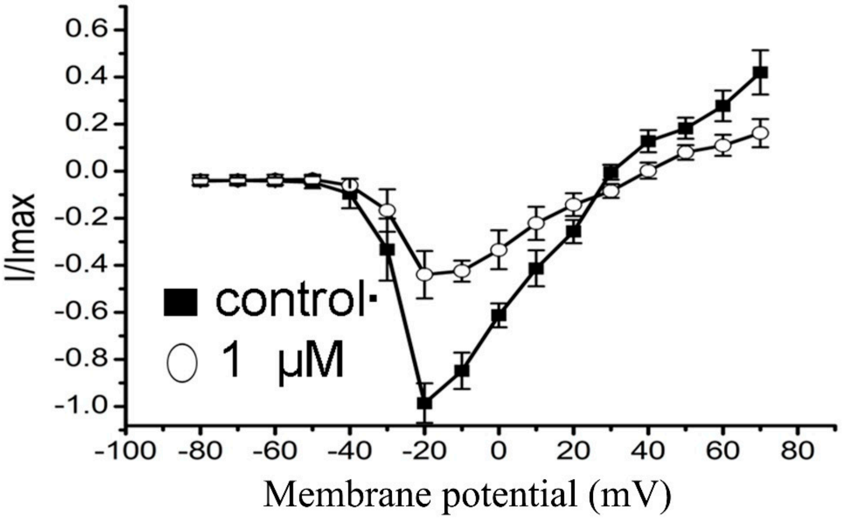
I/Imax
0.6
0.4
0.2
0.0
-0.2
-0.4
-0.6
-0.8
-1.0
-100 -80 -60 -40 -20 0 20 40 60 80
Membrane potential (mV)
control
1 μM

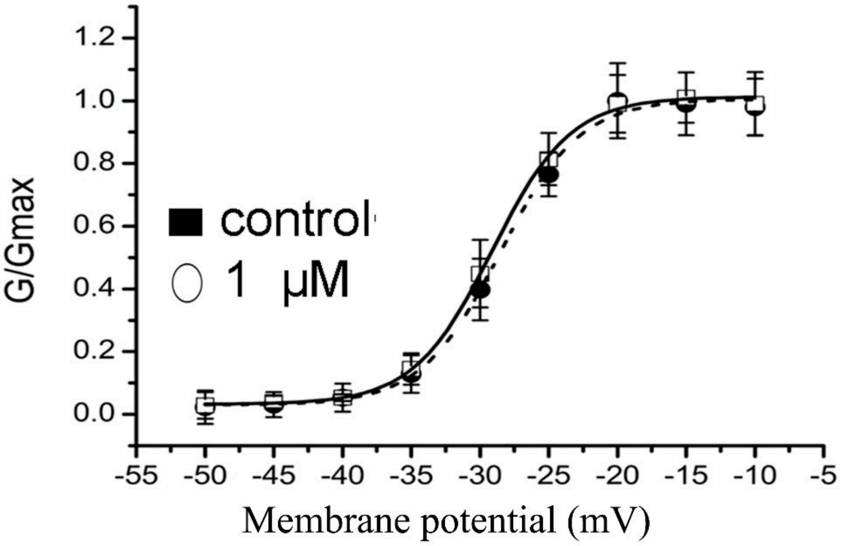
G/Gmax
1.2
1.0
0.8
0.6
0.4
0.2
0.0
-55 -50 -45 -40 -35 -30 -25 -20 -15 -10 -5
Membrane potential (mV)
control
1 μM

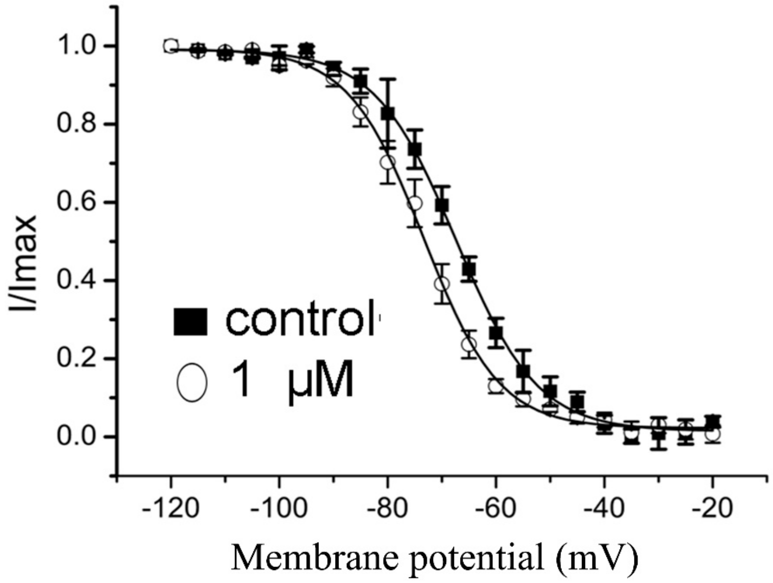
I/Imax
1.0
0.8
0.6
0.4
0.2
0.0
-120 -100 -80 -60 -40 -20
Membrane potential (mV)
control
1 μM

Figure S3. Effects of JZTX-34 on the current-voltage relationship and the steady-activation and inactivation of sodium channel subtypes Nav1.7. (**A**) Effect of 1 μM toxin on the current-voltage relationship of sodium currents. Cells were held at −80 mV, and sodium currents were induced by 50 ms depolarizing steps to various potentials ranging from −80 to +80 mV with 10 mV increments. (**B**) Effect of 1 μM toxin on the steady-activation of Nav1.7. The steady-activation kinetics was estimated based on the data from A. The conductance was calculated using the equation $G(\mathrm{Nav}) = I/(V\text{-}Vrev)$ in which *I*, *V*, *Vrev* represent inward current value, membrane potential, and reversal potential, respectively. Data were plotted as a fraction of the maximum conductance. (**C**) Effect of 1μM toxin on steady-state inactivation of Nav1.7. The voltage dependence of steady-state inactivation was estimated using a standard double pulse protocol in which sodium currents were induced by a 20 ms depolarizing potential of -10mV following a 500 ms prepulse at potentials that ranged from −100 mV to −10 mV. Currents were plotted as a fraction of the maximum peak current. Data points (mean±S.E.) were fitted according to the boltzmann equation (n=5).
